# Growth Hormone Improves Nerve Regeneration, Muscle Re-innervation, and Functional Outcomes After Chronic Denervation Injury

**DOI:** 10.1038/s41598-019-39738-6

**Published:** 2019-02-28

**Authors:** Joseph Lopez, Amy Quan, Joshua Budihardjo, Sinan Xiang, Howard Wang, Kiron Koshy, Christopher Cashman, W. P. Andrew Lee, Ahmet Hoke, Sami Tuffaha, Gerald Brandacher

**Affiliations:** 1 Department of Plastic & Reconstructive Surgery, Johns Hopkins University School of Medicine, Baltimore, MD USA; 2 Department of Neuroscience, Johns Hopkins University, Baltimore, MD USA

## Abstract

This study investigates the efficacy of systemic growth hormone (GH) therapy in ameliorating the deleterious effects of chronic denervation (CD) injury on nerve regeneration and resulting motor function. Using a forelimb CD model, 4 groups of Lewis rats were examined (n = 8 per group): *Group-1 (negative control)* 8 weeks of median nerve CD followed by ulnar-to-median nerve transfer; *Group-2 (experimental)* 8 weeks of median nerve CD followed by ulnar-to-median nerve transfer and highly purified lyophilized pituitary porcine GH treatment (0.6 mg/day); *Group-3 (positive control)* immediate ulnar-to-median nerve transfer without CD; *Group-4 (baseline)* naïve controls. All animals underwent weekly grip strength testing and were sacrificed 14 weeks following nerve transfer for histomorphometric analysis of median nerve regeneration, flexor digitorum superficialis atrophy, and neuromuscular junction reinnervation. In comparison to untreated controls, GH-treated animals demonstrated enhanced median nerve regeneration as measured by axon density (p < 0.005), axon diameter (p < 0.0001), and myelin thickness (p < 0.0001); improved muscle re-innervation (27.9% vs 38.0% NMJs re-innervated; p < 0.02); reduced muscle atrophy (1146 ± 93.19 µm^2^ vs 865.2 ± 48.33 µm^2^; p < 0.02); and greater recovery of motor function (grip strength: p < 0.001). These findings support the hypothesis that GH-therapy enhances axonal regeneration and maintains chronically-denervated muscle to thereby promote motor re-innervation and functional recovery.

## Introduction

The length of time that elapses prior to reinnervation is the most important factor contributing to poor outcomes following peripheral nerve injury. It is well-known that denervated muscle undergoes atrophic changes involving permanent loss of myofibrils and motor endplates^1,2^, and the degree of atrophy increases with the duration of denervation^3,4^. Furthermore, proliferating Schwann Cells (SCs) within the distal traumatized nerve that lack axonal interaction will eventually lose the capacity to secrete neurotrophic factors and maintain the bands of Bungner; this process of denervation-induced SC senescence has been shown to greatly impair axonal regeneration^5–7^.

Given the importance of prompt reinnervation, much attention has been directed towards developing therapies to accelerate axonal regeneration, and a number of experimental agents have demonstrated efficacy in this regard^8–10^. In contrast to other therapeutics that have been investigated, growth hormone (GH) has the potential to speed axonal regeneration and also maintain denervated muscle and SCs prior to reinnervation^11^. GH is released by the pituitary gland in response to growth hormone releasing hormone (GHRH) stimulus from the hypothalamus. GH exerts its action primarily by stimulating synthesis of insulin-growth factor-1 (IGF-1) from the liver and peripheral tissues, and to a lesser extent by direct action on several tissues^12^. IGF-1 plays an important role in neuronal survival and regeneration and has been shown to stimulate neurite outgrowth, *in vitro*^13,14^. IGF-1 has also been shown to markedly reduce the rate of denervation-induced muscle atrophy^15–17^ and stimulate axonal sprouting into denervated muscle during the process of reinnervation^18^. Furthermore, IGF-1 is upregulated by SCs following nerve injury^19^ and has been shown to enhance SC survival, proliferation, mobilization and myelinating capacity^20–23^.

Translational studies in rodents performed by our group and others have demonstrated that GH therapy can improve nerve regeneration and muscle reinnervation following *acute* nerve injury repair^24–26^. However, a model in which chronic denervation is induced prior to nerve repair is needed to fully assess the hypothesized multi-modal mechanism of action of GH therapy involving maintenance of denervated muscle and SCs, in addition to direct neurotrophic effects on regenerating axons.

In this study, we used a rat median nerve chronic denervation (CD) model to investigate the ability of GH therapy to reduce denervation-induced muscle atrophy and SC senescence, enhance axonal regeneration and muscle re-innervation, and thereby improve functional recovery.

## Results

### Median Nerve Chronic Denervation and Repair Model

#### Median Nerve Histomophometry

**Figure 1:**
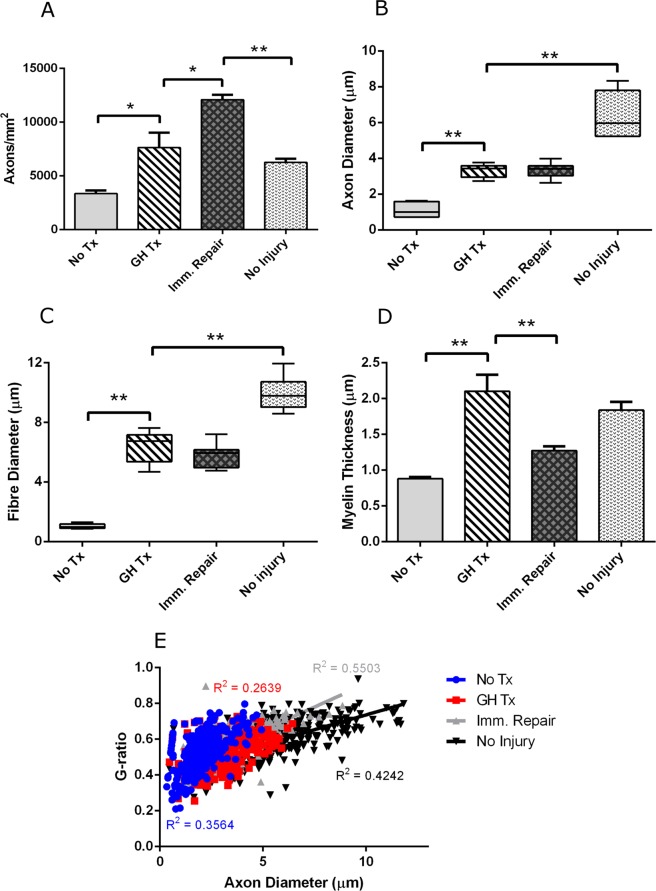
GH therapy improves median nerve regeneration. (**A**) Total axon count (**B**) Mean axon diameter (**C**) Fibre diameter and (**D**) Myelin thickness. (**E**) Median nerve g-ratios as a function of axon diameter. Error bars represent standard error. *p < 0.05, **p < 0.001. Abbrev: GH = growth hormone.

Histom-orphometric analysis of regenerating axons within the distal median nerve demonstrated greater total number of axons (7627 ± 1389 vs 3348 ± 283.6 vs; *p* = 0.0046) greater axon diameter (3.32 ± 0.1540 vs 1.115 ± 0.1463; *p* < 0.0001), greater myelin thickness (2.102 ± 0.2308 vs 0.8788 ± 0.0213; *p* < 0.0001), and greater fiber (axon plus myelin) diameter (6.397 ± 0.4357 vs 1.025 ± 0.0559; *p* < 0.0001), in GH-treated animals in comparison to untreated controls. The GH-treated animals demonstrated fewer total number of axon (7627 ± 1389 vs. 12080 ± 458.2; *p* = 0.0051) and lesser myelin thickness (2.102 ± 0.2308 vs. 1.273 ± 0.0584; *p* = 0.0018) than positive controls with immediate repair, but no significant differences in fiber diameter (6.397 ± 0.4357 vs. 5.786 ± 0.2829; p = 0.2429) or axon diameter (3.32 ± 0.1540 vs. 3.349 ± 0.1475; *p* = 0.8966) were observed. Finally, the GH-treated animals demonstrated similar g-ratio to untreated animals (0.5286 ± 0.0110 vs. 0.520 ± 0.0244; *p* = 0.7419). GH-treated animals demonstrated a lower g-ratio when compared to positive controls with immediate repair (0.5286 ± 0.0110 vs. 0.55763 ± 0.0121; *p* = 0.0449) (Fig. 1).

#### Percent Reinnervation of Neuromuscular Junctions

**Figure 2:**
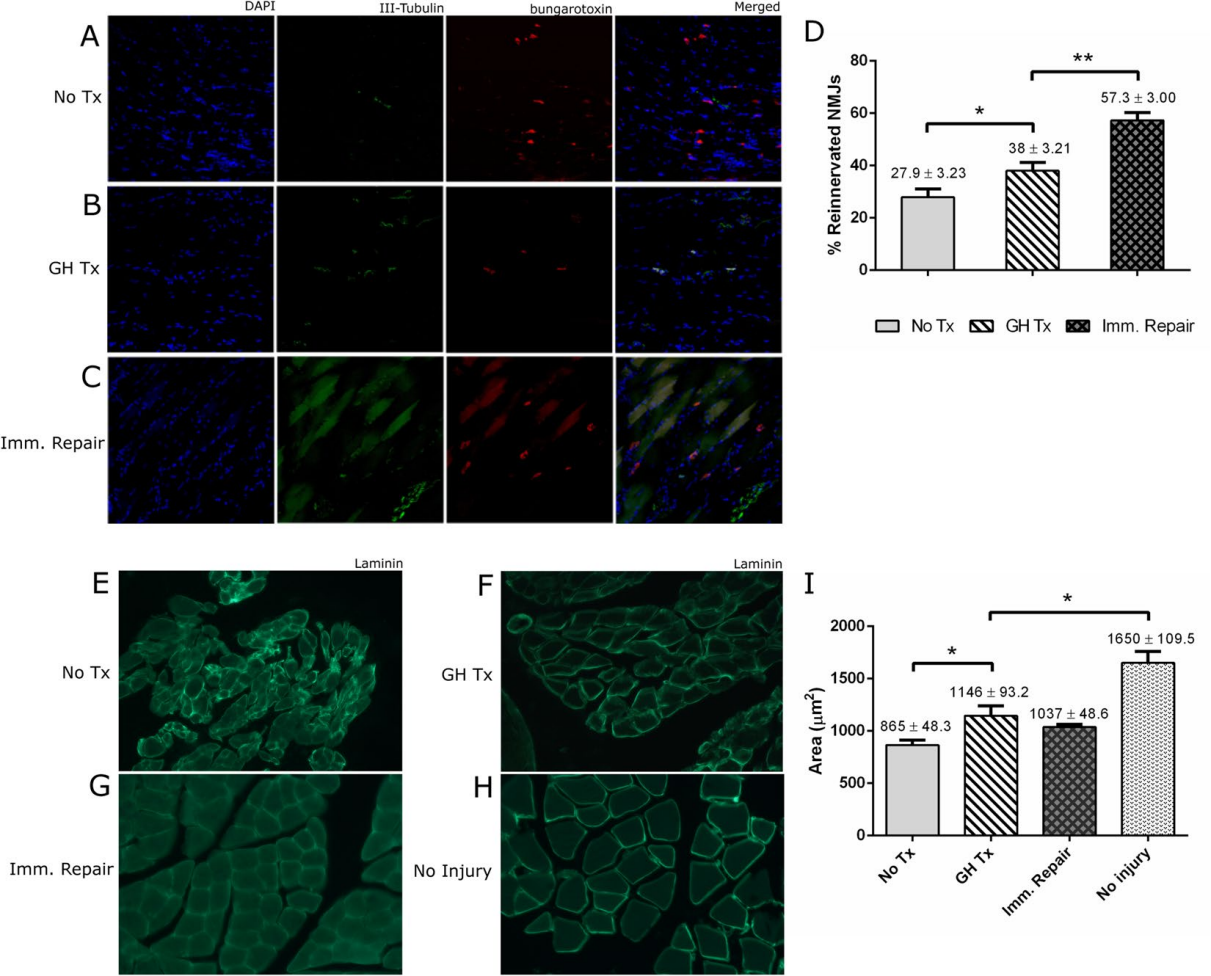
GH therapy improves neuromuscular innervation and reduces muscle atrophy. (**A**–**C**) Representative confocal images (20x) of median-nerve innervated flexor muscles immunostained for DAPI (blue), α-β-III-Tubulin (green), and α-bungarotoxin (red). (**D**) Quantification of the total number of the motor endplates and the number of co-stained reinnervated motor endplates. (**E**–**H**) Representative confocal images (20x) of median-nerve innervated flexor muscle immunostained for laminin. I) Quantification of the cross-sectional muscle area.

GH-treated animals demonstrated significantly greater percent reinnervation of neuromuscular junctions in the flexor digitorum superficialis muscle in comparison to untreated controls (38.0 ± 3.210% vs 27.9 ± 3.230%; *p* = 0.0281). GH-treated animals demonstrated less percent reinnervation of neuromuscular junctions in comparison to positive controls with immediate repair (38.0 ± 3.210 vs 57.30 ± 3.0; *p* < 0.0001). (Fig. 2A–D).

#### Flexor Digitorum Superficialis Myofiber Cross-Sectional Area and Wet Muscle Mass

GH-treated animals demonstrated greater myofiber cross-sectional area in comparison to untreated controls (1146 ± 93.19 µm^2^ vs 865.2 ± 48.33 µm^2^; *p* = 0.0233). No significant difference was noted between GH-treated animals and immediate repair positive controls (1146 ± 93.19 µm^2^ vs 1037 ± 26.58 µm^2^; p = 0.3303) (Fig. 2E–I).

#### Electrophysiologic Assessments

**Figure 3:**
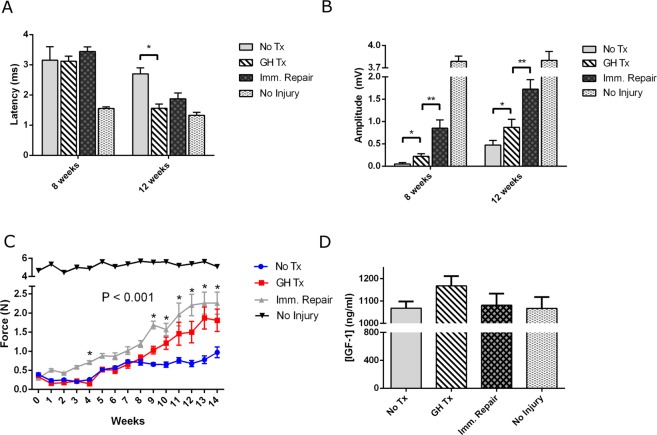
GH therapy augments forelimb function (**A**) Experimental CMAP latency at 8 and 12 weeks post-repair. (**B**) Experimental CMAP amplitudes at time points 8 and 12 weeks post-repair. (**C**) Post-operative functional grip strength until the end-point at 14 weeks post-repair. (**D**) Circulating serum levels of IGF-I were measured via ELISA. Bars represent standard error. *p < 0.05, **p < 0.001. Abbrev: GH = growth hormone; CMAP = compound muscle action potential.

GH-treated animals displayed greater compound muscle action potentials (CMAP) amplitudes at weeks 8 and 12 (8 weeks: 0.220 ± 0.59 mV vs 0.050 ± 0.33 mV, *p* = 0.02; 12 weeks: 0.871 ± 0.178 mV vs 0.475 ± 0.100 mV, *p* = 0.05) and decreased CMAP latency at week 12 following median nerve repair (1.563 ± 0.139 ms vs 2.700 ± 0.199 ms, *p* = 0.0005) in comparison to untreated controls. GH-treated animals demonstrated diminished CMAP amplitude at weeks 8 and 12 (8 weeks: 0.220 ± 0.59 mV vs 0.85 ± 0.185 mV, *p* = 0.0092; 12 weeks: 0.871 ± 0.178 mV vs 1.725 ± 0.210 mV, *p* = 0.0093) and greater CMAP latency at week 12 (1.563 ± 0.139 ms vs 1.875 ± 0.193 ms, *p* = 0.0005) in comparison to positive controls with immediate repair (Fig. 3A,B).

#### Grip Strength Testing

GH-treated animals displayed greater recovery of grip strength than untreated controls at 9–14 weeks following median nerve repair (1.03 ± 0.10 N vs 0.66 ± 0.04 N, *p* = 0.004; 1.21 ± 0.16 N vs 0.65 ± 0.07 N, *p* = 0.006; 1.46 ± 0.30 N vs 0.76 ± 0.07 N, *p* = 0.0394; 1.50 ± 0.28 N vs 0.68 ± 0.08 N, *p* = 0.0137; 1.87 ± 0.29 N vs 0.78 ± 0.10 N, *p* = 0.0032; 1.81 ± 0.29 N vs 0.97 ± 0.14, *p* = 0.021, respectively). There were no statistically significant differences in grip strength between GH-treated animals and positive controls with immediate repair from weeks 10 through 14 following median nerve repairs (p > 0.05) (Fig. 3C).

#### Systemic IGF-1 Levels

As measured by enzyme-linked immunosorbent assay (ELISA), GH-treated animals displayed no significant difference in circulating, serum levels of IGF-I than untreated controls after 8 weeks of GH therapy. However, a trend towards elevated IGF-I levels in GH-treated animals was noted (1168 ± 44.0 ng/ml vs 1068 ± 29.9 ng/ml, *p* = 0.0863) (Fig. 3D).

### Sciatic Nerve Chronic Denervation Without Repair Model

#### Gastrocnemius Myofiber Cross-Sectional Area and Wet Muscle Mass

**Figure 4:**
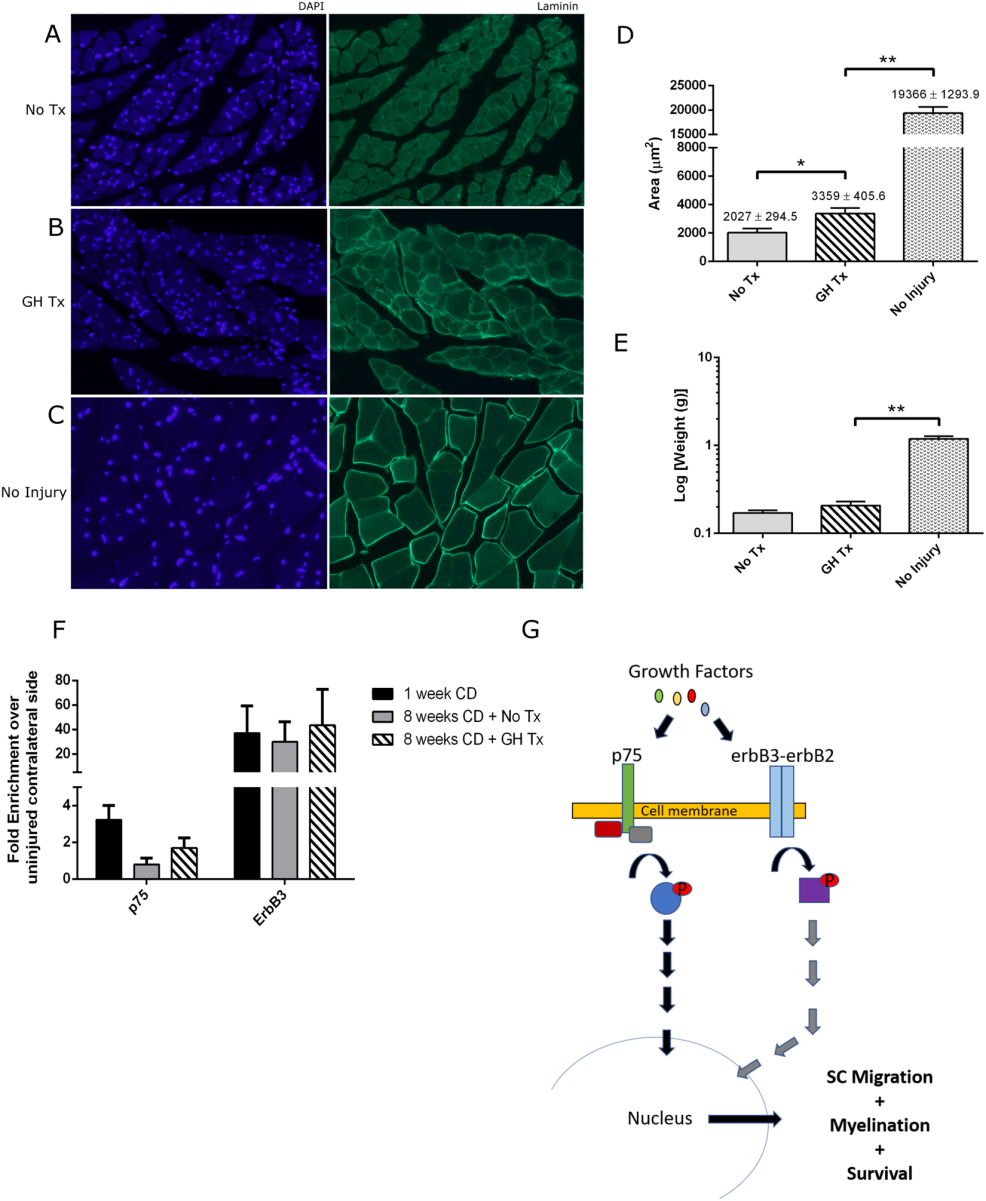
GH therapy reduces muscle atrophy but does not augment SC proliferation after 8 weeks of treatment. (**A**–**C**) Representative confocal images (20x) of gastrocnemius muscle fibers immunostained for DAPI (blue) and laminin (green). (**D**) Quantification of the cross-sectional muscle area (**E**) Quantification of gastrocnemius muscle weight. (**F**) SC expression of p75 and erbB3. Expression levels were determined by RT-PCR analyses in mRNA prepared from SC extracted from nerves in experimental and control groups. All expression levels were calibrated to the animal’s contralateral naïve (non-injured) sciatic nerve. TBP was used as the housekeeping gene. (**G**) Visual representation of the molecular pathways activated by cell surface receptors, erbB and p75, and their involvement in SC proliferation and activation. Bars represent standard error. *p < 0.05, **p < 0.001. Abbrev: GH = growth hormone; SC = Schwann cells.

GH-treated animals demonstrated greater myofiber cross-sectional area in comparison to untreated controls (3359 ± 405.6 µm^2^ vs 2027 ± 294.5 µm^2^, *p* = 0.0258) (Fig. 4A–D). No significant difference in wet muscle mass was noted between these groups (Fig. 4E).

#### Markers for SC Proliferation and Viability

No significant differences in p75 and erbB3 expression between GH-treated animals and untreated controls were observed, though there was a trend towards elevated expression of these markers in the GH-treated animals (p75: 1.692 ± 0.557 -fold vs 0.802 ± 0.338-fold, *p = *0.19361; erbB3: 43.39 ± 29.51 -fold vs 29.96 ± 16.26-fold, *p* = 0.6960) (Fig. 4F). Similarly, when S100b was used as the housekeeping gene instead of TBP, no significant differences in p75 and erbB3 expression between groups were seen (p75: 1.863 ± 0.718 -fold vs 0.391 ± 0.217-fold, *p = *0.0698; erbB3: 13.29 ± 3.71-fold vs 5.43 ± 1.79-fold, *p* = 0.077) (Supplementary Figure).

## Discussion

This study is the first to assess the effects of GH therapy in a model in which chronic denervation is induced prior to nerve repair. Because the regenerative distances in rat nerve injury models are very short relative to those seen in humans, axons will reach their targets before the effects of prolonged denervation can take place. To better model the deleterious effects of chronic denervation on muscle and distal Schwann Cells (SCs) that occur with human nerve injuries, Gordon, *et al*., developed a rat model in which the tibial nerve is transected and left in discontinuity for a period of time prior to peroneal-to-tibial nerve transfer^4^. Given the limitations in performing serial functional testing with this model, a forelimb chronic denervation model that allows for serial grip strength measurements was used^27,28^.

The beneficial effects of the GH axis on motor, sensory, and sympathetic neurons have been extensively studied^29^. Following nerve trauma, local IGF-I production is upregulated at the site of nerve injury^30^. IGF-1, via the PI3-kinase pathway, upregulates the expression of focal adhesion molecules and GAP43, and these changes promote neurite growth cone motility and prevent neuronal apoptosis^13,31^. In our study, we did not find a statistically significant elevation in serum IGF-1 levels in the GH-treated group; however, there was a clear trend toward elevation in the treated group and we believe this likely represents a type-II error. It is well-known that GH induces upregulation of IGF-1, which in turn serves to mediate the effects of the GH/IGF-1 axis. Of note, we did not collect serum samples at the same time of day in this study, and this likely introduced variability due to the temporal fluctuations in IGF-1 release.

As in prior studies assessing the effects of GH in acute injury repair models^24–26^, we observed enhanced nerve regeneration with GH treatment. While the greater number of fibers observed with axonal histomorphometry distal to the coaptation could be interpreted as resulting from collateral sprouting when viewed in isolation, this finding in combination with greater percent reinnervation of neuromuscular junctions, improved electrophysiologic parameters of regeneration, and greater functional recovery support the conclusion that GH treatment provides meaningful improvement in nerve regeneration. That being said, retrograde labelling would be helpful in future studies to delineate the number of motor neurons contributing to end-organ reinnervation, as motor unit expansion could have partially explained the differences observed in neuromuscular junction reinnervation and functional recovery. In contrast to prior studies, nerve regeneration in our model took place within a chronically denervated pathway. While we hypothesize that a direct treatment effect on regenerating axons likely contributed to the observed improvement, it is unlikely this effect alone could overcome the lack of trophic support within the distal regenerative pathway resulting from chronic denervation (CD). Another explanation is that GH treatment via IGF-1 signaling also served to ameliorate the effects of CD on the proliferating SCs within the distal nerve such that they could continue to provide trophic support to regenerating axons. IGF-1 has been shown to play a crucial role in promoting SC survival, maturation and differentiation to myelinating phenotypes^32–34^. More specifically, IGF-I and II promote SC expression of myelin basic protein and myelin associated glycoprotein via the PI3-K/AKT pathway, and stimulate DNA synthesis and proliferation^35,36^. In our study, we aimed to isolate the effects of GH on chronically denervated SCs by using a model in which the sciatic nerve is transected and left in discontinuity for 8 weeks. We then performed RT-PCR for markers of SC proliferation and viability. While we did not observe statistically significant differences in these markers, we did note trends towards increased expression in GH-treated animals for all markers, approaching significance for erbB3 and p75 expression. We also did not find that the p75 fold enrichment value was significantly increased in the positive control group undergoing one week of denervation, although there was a trend towards increased expression. The lack of statistical significance may therefore have represented a type-II error, with inadequate power to account for the inherent variability in the RT-PCR assay. Further studies are needed to elucidate the effects of GH treatment on denervated SCs, *in vivo*.

It is well understood that prolonged denervation of muscle results in decreased muscle mass and contractility^37^. To counteract the effects of denervation, several mechanistic studies have found that overexpression of IGF-1 can markedly reduce the rate of denervation-induced atrophy through upregulation of MADbx, MuRF1 and the mTOR/AKT pathway^15,16^. Other studies have also shown that IGF-I and IGF-II can stimulate nerve sprouting into denervated muscle during the process of reinnervation^18^. In our study, we observed histologic evidence for decreased muscle atrophy in GH-treated animals, supporting the hypothesis that GH can ameliorate the effects of CD on muscle. In a prior study using a model in which the sciatic nerve was transected and immediately repaired, we also noted decreased atrophy^25^; however, with that model it was not possible to determine whether the decreased atrophy was due to accelerated axonal regeneration and muscle reinnervation arresting atrophy sooner or a direct effect on denervated muscle prior to reinnervation. In contrast, the results from this current study provide stronger evidence for the positive direct effects of GH treatment on muscle. In the median nerve chronic denervation and repair model, the long length of time in which muscle was subjected to denervation prior to reinnervation makes it unlikely that accelerated axonal regeneration alone could account for the diminishment of atrophy noted. In the sciatic nerve transection-without-repair model, the decreased atrophy could only be due to a direct effect of GH treatment on muscle, as axonal regeneration and muscle reinnervation could not have occurred.

Our study is the first to use robust functional assessment to demonstrate enhanced motor recovery with GH treatment. As expected, the animals subject to CD without treatment demonstrated poor functional recovery. In contrast, the animals subjected to CD with GH treatment demonstrated markedly improved recovery of motor function, with grip strength values similar to those observed in animals with immediate nerve repair. We also observed improvements in electrophysiologic parameters of nerve regeneration and motor reinnervation with GH treatment in comparison to untreated controls. These findings are likely explained by the direct trophic effects of IGF-1 on regenerating axons, as well as the amelioration of denervation-induced muscle atrophy and SC senescence. In future studies, it will be important to quantify IGF-I gene expression changes and local tissue concentrations and determine the optimal IGF-1 level and corresponding GH dose that produces maximal response.

In summary, our experiments support the hypothesis that GH can enhance axonal regeneration and also ameliorate the deleterious effects of chronic denervation on muscle to thereby improve motor re-innervation and functional recovery in rodents. Our results regarding maintenance of denervated SCs are equivocal and warrant further investigation. These findings provide further support for clinical translation of this promising treatment modality to promote improved functional recovery following peripheral nerve injury.

## Methods

### Animals

This study was carried out utilizing the *Guide for the Care and Use of Laboratory Animals* of the Nationals Institute of Health (No. 86–23). The protocol was approved by the Johns Hopkins University Animal Care and Use Committee. Animals were monitored by staff twice daily to ensure good health. Regular physical examinations were performed. Surgical sites were monitored for cellulitis, bleeding, abscesses, seroma, and dehiscence. All surgical procedures were conducted under standard sterile conditions. Adult male Lewis Rats, purchased from Charles River Laboratory (Wilmington, MA), were used. All animals were 6–8 weeks old and weighed 160–180 grams. All Animals were randomized into four groups: (1) eight weeks of median nerve chronic denervation injury followed by nerve repair and no therapy (negative control); (2) eight weeks of median nerve chronic denervation followed by nerve repair and GH therapy (experimental); (3) Immediate median nerve repair and no therapy (positive control); (4) Sham surgical intervention and no therapy (baseline control) (Table 1). The contralateral sciatic nerve of rodents in the negative control (group 1), experimental (group 2), and positive control (group 3) groups were transected and left in discontinuity to assess the effects of GH therapy on chronically denervated muscle and SCs that are not reinnervated (see “Sciatic Nerve Chronic Denervation” below and Table 2). Each group consisted of eight animals. Once all rodents reached the final end-point of 14 weeks post-median nerve repair, they were euthanized humanely after proper tissue harvesting.

**Table 1:** Median Nerve CD Injury & Repair Experimental Groups.

Group No.	Group (n = 8)	Length of CD injury	Type	Experiment	Endpoint
1	Median nerve transection followed by CD injury and delayed repair	8 weeks	Negative Control	No treatment	14 weeks post-repair
2	Median nerve transection followed by CD injury and delayed repair	8 weeks	Experimental	Systemic GH therapy^¥^	14 weeks post-repair
3	Median nerve transected followed by immediate repair	0 weeks	Positive Control	No treatment	14 weeks post-repair
4	Median nerve with no injury (sham surgery)	0 weeks	Baseline	No treatment	14 weeks post-surgery

^¥^Systemic GH therapy consisted of twice daily subcutaneous back injections of highly purified lyophilized pituitary porcine GH (0.6 mg/day) throughout the chronic denervation period (8 weeks) and post-repair period (14 weeks). Abbrev: CD = Chronic Denervation; GH = Growth Hormone.

**Table 2:** Contralateral Lower Extremity Sciatic Nerve CD and Experimental Groups.

Group (n = 8)	Length of CD injury	Experiment	Endpoint
Sciatic nerve transection followed by CD injury	8 weeks	No treatment	8 weeks
Sciatic nerve transection followed by CD injury	8 weeks	Systemic GH Therapy	8 weeks
Sciatic nerve transection followed by CD injury	1 week	No Treatment	1 week
Sciatic nerve transection (naïve)	None	No Treatment	8 weeks

All lower extremity contralateral limbs served as internal experimental groups to assess the effects of GH on SC proliferation/senescence (via qPCR) in chronically-denervated sciatic nerves and gastrocnemius muscle atrophy. As presented in Table 1, systemic GH therapy consisted of twice daily subcutaneous back injections of highly purified lyophilized pituitary porcine GH (0.6 mg/day) throughout the chronic denervation period. Abbrev: GH = Growth Hormone; CD = chronic denervation.

### Median Nerve Chronic Denervation Model

**Figure 5:**
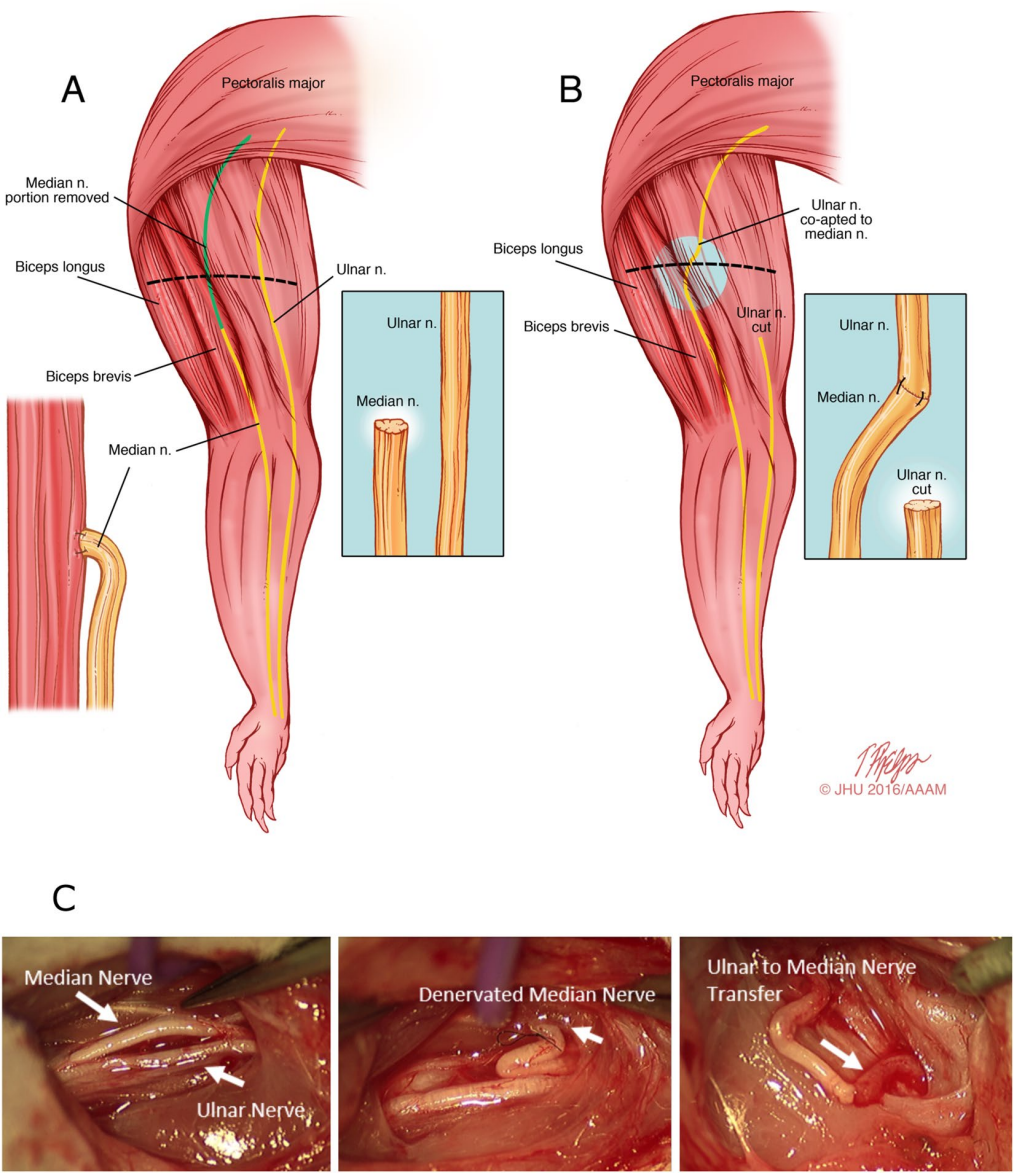
Median nerve-based CD model. (**A**) The median nerve is transected and denervated for a given period (e.g. 8 weeks in our study). To prevent nerve regeneration across the transected median nerve throughout the CD period, ~1.5 cm of the proximal median nerve stump is resected and the distal stump is sutured to the biceps brevis. (**B**) In order, to isolate the effects of chronic denervation from chronic axotomy injury, the distal, denervated median nerve stump is co-apted to a the freshly transected ulnar nerve at the time of repair. (**C**) *In-vivo* presentation of the ulnar to median nerve transfer at the time of repair.

We used a rodent forelimb chronic denervation model that provides dependable assessment of behavioral functional recovery using grip strength testing (see “Functional Measurements of Muscle Power” below). In this model, the median nerve is first transected and left in discontinuity for 8 weeks to allow for chronic denervation to take place (Fig. 5). Then, the distal, chronically denervated median nerve stump is co-apted to the freshly transected proximal ulnar nerve at the time of repair. Thereafter, functional and electrophysiologic parameters of recovery are measured weekly tools (see below).

### Surgical Details

In stage one, a transverse incision is made at the mid-humerus level. The median nerve is exposed and transected 2 mm from biceps aponeurosis. (Group 3 rats undergo sham surgery at this stage, with median nerve exposure but no transection). To avoid regeneration across the transected median nerve, the distal median nerve stump is sutured with two 10-0 epineural sutures to the underside of the biceps brevis muscle, and the proximal median nerve stump is resected as proximally as possible to create a large nerve gap. The surgical site is then closed with sterile suture. Post-operative analgesia consisted of subcutaneous buprenorphine injections at a concentration of 0.05 mg/kg twice daily for 72 hours postoperatively with the first dose administered during surgery.

After 8 weeks of median nerve chronic denervation, a second stage procedure is performed under deep inhalation anesthesia with isoflurane under sterile conditions. The original transverse mid-humerus incision is re-opened and the median and ulnar nerves are exposed. The suture holding the distal stump of the median nerve to the underside of the biceps muscle is used as a landmark for identification. After isolation and dissection of the distally denervated median nerve stump and the intact ulnar nerve, the ulnar nerve is transected as distally as possible, and the proximal ulnar nerve stump is coapted to the the distal median nerve stump with 10-0 epineural sutures. The surgical site is then closed with sterile suture and the animals are returned to their regular housing with adequate analgesia after post-op monitoring.

### Sciatic Nerve Chronic Denervation

To isolate the effects of GH therapy on chronically denervated muscle and Schwann Cells (SCs), independent of the effects of reinnervation, the contralateral sciatic nerve is transected and left in discontinuity at the time of median nerve transection in groups 1, 2, and 3 (Table 2). The sciatic nerve was transected as proximally as possible, to provide as much length of distal, denervated nerve tissue as possible. 8 weeks later, the denervated sciatic nerve and gastrocnemius muscle of rodents were harvested for analysis for groups 1 and 2. In group 3 (positive control), the denervated sciatic nerve was harvested one week following transection to allow for maximal SC proliferation.

### GH Therapy

From the time of median nerve transection until the time of sacrifice, animals were treated with twice daily subcutaneous injections of highly purified lyophilized pituitary porcine GH (300ug/injection) (National Hormone and Peptide Program, Los Angeles, CA)^38^.

### Grip Strength Testing

To assess functional recovery, grip strength testing was performed using the Chatillon force measurement device (Ametek; Largo, FL). Grip strength was measured at baseline (prior to median nerve transection) and every week following median nerve repair until sacrifice. the rat was dangled by its tail to elicit the grasp reflex. To encourage use of the injured forelimb, the contralateral forelimb was immobilized with tape. After grasping a bar attached to a force transducer, the rat was slowly pulled by the tail in a horizontal vector until grasp of the bar was released. Maximum force generated prior to release was measured. Five trials per session were performed and averaged^39^.

#### Evoked Nerve Conduction Studies (NCS)/Compound Muscle Action Potential (CMAP)

To assess electrophysiologic parameters of nerve regeneration and muscle reinnervation, CMAP studies were performed prior to surgery, and at 8 and 12 weeks after median nerve repair. The median nerve was stimulated proximal to the injury site at the deltoid tuberosity with a bipolar subdermal needle electrode (CareFusion, Middleton, WI), and the recordings were carried out with PowerLab (AD Instruments, Colorado Springs, CO). Outcome measures included latency and amplitude of compound muscle action potentials.

#### Nerve Histomorphometry (NH)

To measure the extent of axonal regeneration, NH was performed. The median nerve was harvested at time of sacrifice, fixed in 2% glutaraldehyde, post-fixed with 1% osmium tetroxide, and embedded in Araldite® 502 (Polyscience). A 10-mm segment of median nerve, 5 mm distal to the repair site (at the level of the rodent wrist) was cross-sectioned and stained with 1% toluidine blue for light microscopy. Digital images of the median nerve were then taken using an unbiased sampling method of non-overlapping regions of the entire cross-section. Total number, density, diameter, and g-ratio of myelinated axons was quantified using Image J software. For each sample, a minimum of 200 myelinated axons was measured and the average was counted as n = 1.

#### Neuromuscular Junction Analysis

To measure the extent of muscle reinnervation, forearm extrinsic finger flexor muscles (innervated by the median nerve) are harvested at sacrifice, longitudinally sectioned and stained with primary antibody anti-TUJ-1 (against neurofilament; 1:2000, Biolegend, MRB-435P), secondary Flourescein G anti-rabbit (1:800, Ventor, FI-100), and α-bungaratoxin (against motor endplate; 1:1000, Invitrogen, B-13423). Immunofluorescence microscopy was used to count total number of the motor endplates and the number of co-stained reinnervated motor endplates. The percent of reinnervated motor endplates was then calculated. For each sample, a minimum of 50 non-overlapping regions of the entire cross-section of muscle were counted.

#### Muscle Atrophy Analysis

To measure the extent of muscle atrophy, the forearm extrinsic finger flexor muscles were harvested at sacrifice, fixed in 4% paraformaldehyde, embedded in OCT, frozen, sectioned transversely and stained using primary antibody against laminin-γ1 (Chemicon, Billerica, MA). Following application of secondary antibody, immunofluorescent images were captured and myofiber cross-sectional area was quantified using Image J software. The diameters of 700–1000 fibers per animal were measured. Average from each animal was counted as n = 1. Prior to processing, the wet muscle mass was measured.

### IGF-I Serum Enzyme-linked Immunosorbent Assay (ELISA)

To measure the circulating levels of IGF-I expression in animal serum, serum was collected from the tails of rats at 8 weeks post-GH therapy. The ELISA was performed per the commercial IGF-I rat/mouse quantikine ELISA kit instructions (MG100, R&D Systems, USA). In brief, 50ul of serum was mixed with 50uL of assay diluent in a 96-well plate and incubated for 2 hours at room temperature. The supernatant was aspirated, and the wells were serially washed with wash buffer. A substrate solution was then added to each reaction and incubated at room temperature while in the dark for 30 min. Lastly, the reaction was terminated by adding 100ul of stop solution. Utilizing OD 570 as a reference, absorbance at 450 nm was recorded using a TECAN infinite 200 PRO (Tecan, USA).

#### Sciatic Nerve qPCR for Markers of Schwann Cell Denervation

To assess for SC viability after chronic denervation, a portion of the distal denervated sciatic nerve was assessed via qPCR for the following markers: p75 (F 5′-GATTCTAGGGATGTCCTCTG-3′; R 5′-CATCGGAGAATGTAACACTG-3′); ErbB3 (F 5′-AATCTGGACTTCCTCATCAC-3′; R 5′-TTTAGGTAACCTGTGATCTCC-3′); TATA binding protein (a housekeeping gene abbreviated as TBP; F 5′CATCATGAGAATAAGAGAGCC-3′; R 5′-GGATTGTTCTTCACTCTTGG-3′) and S100b (a secondary housekeeping gene abbreviated as S100; F 5′CATCAGTATTCAGGGAGAGAG-3′; R 5′-ACTTCCTGCTCTTTGATTTC-3′). All the primers used in the qPCR studies were from MilliporeSigma as part of the KiCqStart primer offering (MilliporeSigma KSPQ12012) with in silico validation. Additionally, the primers were validated *in vitro* for single band amplification from cDNA only. Absence of gDNA amplification was confirmed by cDNA templates with and without reverse transcriptase. Melting curves of the final qPCR reactant confirmed single peaks. The nerve sample was flash frozen in liquid nitrogen and a TRIzol®-based protocol was used for RNA extraction. Synthesis of cDNA proceed through use of the QuantiTect Reverse Transcription Kit (Qiagen, Valencia, CA). Lastly, qPCR was performed with the QuantiTect SYBR Green Kit (Qiagen, Valencia, CA). TBP was used as the primary housekeeping gene to normalize qPCR data using the ddCT method. However, a secondary analysis using S100b as a housekeeping gene was also presented as a supplementary analysis.

### Statistical Analysis

All results shown are presented as mean ± SE (standard error). All qPCR, nerve histomorphometry, electrophysiological, ELISA and muscle morphology data were compared using an ANOVA to determine whether overall differences existed across groups. Posthoc comparisons between specific groups were completed with a Student’s t-test with Bonferroni correction. A p-value of < 0.05 was considered significant for all analyses. SPSS statistical analysis software was used for all analyses (IBM Corporation, Armonk, New York).

## Supplementary information

Supplementary Data
