# Supplementary material for: Growth Hormone Improves Nerve Regeneration, Muscle Re-innervation, and Functional Outcomes After Chronic Denervation Injury
Source: Sci Rep. 2019 Feb 28;9:3117. doi: 10.1038/s41598-019-39738-6 (PMC6395714; doi:10.1038/s41598-019-39738-6)

**Growth Hormone Improves Nerve Regeneration, Muscle Re-innervation, and Functional Outcomes After Chronic Denervation Injury**

Joseph Lopez, M.D. M.B.A.^1^; Amy Quan, M.D. M.P.H.^1^; Joshua Budihardjo B.S.^1^; Sinan Xiang, B.S.^1^; Howard Wang M.D.^1^; Kiron Koshy M.B.B.S. B.Sc.^1^; Christopher Cashman, M.D. Ph.D.^2^; WP Andrew Lee M.D.^1^, Ahmet Hoke M.D. Ph.D.^2^, Sami Tuffaha M.D.^1*^; Gerald Brandacher M.D.^1*^

^1^Department of Plastic & Reconstructive Surgery, Johns Hopkins University School of Medicine, Baltimore, MD, USA.

^2^ Department of Neuroscience, Johns Hopkins University, Baltimore, MD, USA

*Designates co-corresponding authorship

**Corresponding Author:**

Gerald Brandacher M.D.

Scientific Director, Vascularized Composite Allotransplantation Laboratory

Department of Plastic & Reconstructive Surgery

Email: brandacher@jhmi.edu

Sami Tuffaha M.D.

Department of Plastic & Reconstructive Surgery

Email: [stuffah1@jhmi.edu](mailto:stuffah1@jhmi.edu)

**Supplementary Figure. SC expression of p75 and erbB3.**

Expression levels for both p75 and erbB3 were determine by RT-PCR analyses in mRNA prepared from SC extracted from nerve in experimental and control groups. All expression levels were calibrated to the animal’s contralateral naïve (non-injured) sciatic nerve. S100b was used as the housekeeping gene.


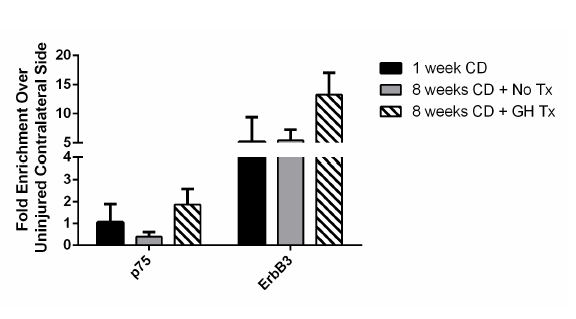

Supplement: Supplementary file 1 — Supplementary Data [file 41598_2019_39738_MOESM1_ESM.docx]
